# A New Biomarker Profiling Strategy for Gut Microbiome Research: Valid Association of Metabolites to Metabolism of Microbiota Detected by Non-Targeted Metabolomics in Human Urine

**DOI:** 10.3390/metabo13101061

**Published:** 2023-10-09

**Authors:** Sijia Zheng, Lina Zhou, Miriam Hoene, Andreas Peter, Andreas L. Birkenfeld, Cora Weigert, Xinyu Liu, Xinjie Zhao, Guowang Xu, Rainer Lehmann

**Affiliations:** 1 CAS Key Laboratory of Separation Science for Analytical Chemistry, Dalian Institute of Chemical Physics, Chinese Academy of Sciences, Dalian 116023, China; zhengsj@dicp.ac.cn (S.Z.); zhouln@dicp.ac.cn (L.Z.); liuxy2012@dicp.ac.cn (X.L.); xj_zhao@dicp.ac.cn (X.Z.); 2 University of Chinese Academy of Sciences, Beijing 100049, China; 3 Institute for Clinical Chemistry and Pathobiochemistry, Department for Diagnostic Laboratory Medicine, University Hospital Tübingen, 72076 Tuebingen, Germany; miriam.hoene@med.uni-tuebingen.de (M.H.); andreas.peter@med.uni-tuebingen.de (A.P.); cora.weigert@med.uni-tuebingen.de (C.W.); 4 Institute for Diabetes Research and Metabolic Diseases of the Helmholtz Zentrum München at the University of Tübingen, 72076 Tübingen, Germany; andreas.birkenfeld@med.uni-tuebingen.de; 5 German Center for Diabetes Research (DZD), 90451 Neuherberg, Germany; 6 Internal Medicine 4, University Hospital Tuebingen, 72076 Tuebingen, Germany

**Keywords:** microbiome, gut flora, metabolomics, metabolites, urine, diagnosis, profiling, gut microbiota

## Abstract

The gut microbiome is of tremendous relevance to human health and disease, so it is a hot topic of omics-driven biomedical research. However, a valid identification of gut microbiota-associated molecules in human blood or urine is difficult to achieve. We hypothesize that bowel evacuation is an easy-to-use approach to reveal such metabolites. A non-targeted and modifying group-assisted metabolomics approach (covering 40 types of modifications) was applied to investigate urine samples collected in two independent experiments at various time points before and after laxative use. Fasting over the same time period served as the control condition. As a result, depletion of the fecal microbiome significantly affected the levels of 331 metabolite ions in urine, including 100 modified metabolites. Dominating modifications were glucuronidations, carboxylations, sulfations, adenine conjugations, butyrylations, malonylations, and acetylations. A total of 32 compounds, including common, but also unexpected fecal microbiota-associated metabolites, were annotated. The applied strategy has potential to generate a microbiome-associated metabolite map (M3) of urine from healthy humans, and presumably also other body fluids. Comparative analyses of M3 vs. disease-related metabolite profiles, or therapy-dependent changes may open promising perspectives for human gut microbiome research and diagnostics beyond analyzing feces.

## 1. Introduction

The gut microbiota very closely interacts with its human host and influences human health [1]. A continuously increasing number of reports show an important role of the gut microbiome in disease development, but also for recovery from diseases, for remission, as well as for disease prevention [2,3,4,5]. Consequently, the luminal (fecal) and mucosal gut microbiota has been intensively investigated in animal models and humans in a comprehensive manner, applying various omics approaches [6]. These studies are first and foremost performed in feces [6,7]. As a result, a tremendous increase in knowledge has been achieved, for instance, about nutritional effects on the microbiome, the pathophysiological consequences of a disbalance of bacterial phyla (e.g., in metabolic diseases), and the role of distinct bacterial species in health and diseases [4,7,8,9,10,11].

These investigations were paralleled by efforts to detect and identify compounds involved in the crosstalk between the gut microbiota and human cells, tissues, or organs [12,13,14,15]. Comprehensive investigations of these metabolites have just started [16,17,18]. Most of the reported metabolites were studied in feces and blood, and just a few in urine [19,20,21,22,23].

Urine is a non-invasively collected sample material. It stands in direct connectivity to blood, since metabolites from the gut microbiome passing the intestinal wall can be transported via the splanchnic bed and the mesenteric veins to the liver, and then partially filtered blood passes through the hepatic veins into the systemic circulation, including the kidneys. Consequently, it could be a useful and easy-to-collect biospecimen to study gut microbiome-associated metabolites. However, the unequivocal linking of a detected metabolite in a body fluid, like blood or urine, to the gut microbiota is quite challenging.

Aiming to contribute to the efforts of closing this gap in knowledge, we hypothesized that a bowel evacuation would change the levels of metabolites associated with the fecal microbiota, thereby enabling the detection of these compounds in human urine. To test this hypothesis, in the current study, we investigated urine samples collected before and after a bowel evacuation, applying a comprehensive, non-targeted, and modifying group-assisted metabolomics approach.

## 2. Materials and Methods

### 2.1. Study Design

The first experiment was performed over 10 days, collecting urine samples at 40 time points before and after a bowel evacuation in an individual self-experiment (n = 1), as well as during the same period after starting again to consume food. Bowel evacuation, as preparation for a colonoscopy as a healthcare check, was achieved using CitraFleet^®^ (sodium picosulfate, sodium citrate) and Tirgon^®^ (Bisacodyl), both from Recordati Pharma (Ulm, Germany). Propofol anesthesia was applied during the colonoscopy. A procedure-related 48 h fasting period was included. Therefore, as a control, a similar experiment was performed, including 48 h of fasting but without bowel evacuation. This experiment ran for 9 days and 30 urine samples were collected. Both urine sample sets consisted of the 1st and 2nd morning urine, as well as spot urine, since urine samples were collected at various time points during the day and night. In a subsequent experiment, 6 healthy volunteers (age: 25–56 years; two females and four males) performed the same bowel evacuation with a total fasting period of 24 h before refeeding (12 h bowel evacuation and a preceding fasting period of 12 h). Urine samples were collected at 4 time points. The first urine sample was taken immediately after waking up at 7.00 am after a 12 h overnight fasting period (1st morning urine). The second sample was the 2nd morning urine, collected at 8.00 am, directly before the start of the bowel evacuation. The third and fourth samples were collected 10 h and 12 h after bowel evacuation, respectively. Furthermore, three out of the six volunteers (age: 25–56 years; one female and two males) performed in addition a 24 h “fasting-only” experiment, as a control. All urine samples were stored at −80 °C until further processing and analyses. The study was conducted according to the Declaration of Helsinki of 1964 and its later amendments. The ethics committee of the University of Tuebingen approved the protocol (188/2017BO2). All volunteers provided written informed consent before the start of the study.

### 2.2. Sample Preparation

Urine was thawed on ice, vortexed, and an internal standard (IS) mix was added (*v*/*v*, 1:10) containing the following 13 ISs: carnitine C2:0-d3, carnitine C6:0-d3, carnitine C10:0-d3, leucine-d3, phenylalanine-d5, tryptophan-d5, cholic acid-2,2,4,4-d4, chenodeoxycholic acid-2,2,4,4-d4, leucine enkephalin, indoxyl sulfate-[^13^C_6_], L-valine-d8, sodium-2-hydroxybutyrate-2,3,3-d3, and L-4-hydroxyphenyl-d4-alanine (details in Table S1). After the addition of the IS-mix, the samples were vortexed again, centrifuged at 18,000× *g* (4 °C for 10 min), and subsequently 100 μL of the supernatant was evaporated. For mass spectrometric analysis, samples were dissolved in 300 µL water:acetonitrile (*v*/*v*, 95:5).

### 2.3. Metabolites Profiling by Liquid-Chromatography Mass Spectrometry (LC-MS)

Non-targeted profiling was performed with an Ultra Performance Liquid Chromatography system (UPLC, Waters Corporation, Manchester, UK) coupled to a Triple TOF 5600+ mass spectrometer (AB SCIEX, Framingham, MA, USA). Chromatographic separation was performed using ACQUITY HSS T3 column (2.1 × 100 mm, 1.8 μm, Waters, Milford, MA, USA). The mobile phases were water (A) and acetonitrile (B) acidified by 0.1% formic acid, respectively. The flow rate was 0.35 mL/min and the total run time was 26 min. The elution gradient initiated with 5% B for 1 min, linearly increased to 50% B at 18 min, then increased to 100% B after 0.5 min, maintained for 4 min, then went back to 5% B after 0.5 min and maintained for 3 min for post-equilibrium. The injection volume was 5 μL. Column temperature was set at 40 °C.

For the MS instrument, full MS-ddMS^2^ mode was used with mass ranges of *m*/*z* 50–1000 Da and 30–1000 Da, respectively. Accumulation times for full scan and ddMS^2^ acquisition modes were 0.25 s and 0.03 s, respectively. Cycle time was 0.75 s. Declustering potential was set at 90. Both electrospray positive ion (ESI^+^) and negative ion (ESI^−^) modes were used. Electrospray voltages were set at 4.5 kV for ESI^+^ mode and −4.0 kV for ESI^−^ mode; ionspray temperature was set at 500 °C; ion source gas1 was 50 psi; ion source gas2 was 50 psi; and curtain gas was 35 psi. For dd-MS^2^, collision energies of 15 V, 30 V, and 45 V were applied and MS/MS fragmentation patterns of the 15 most intense ions in full scan were acquired. Every sixth sample was followed by a quality control (QC) analysis of pooled urine.

### 2.4. Data Processing

Peak detection and alignment were conducted by MarkerView software 1.2.1(AB SCIEX, Framingham, MA, USA). The parameters for peak detection were as follows: minimum spectral peak width of 10 ppm, minimum retention time (t_R_) peak width of 5 scans, and noise threshold of 1000. For peak alignment, a t_R_ tolerance of 0.5 min and mass tolerance of 10 ppm were used. After applying “modified 80% rule” to remove missing values and then removing isotope ions [24], the intensity of each peak was normalized to an appropriate IS. Only peaks with relative standard deviation (RSD) of responses in QC samples less than 30% were kept for subsequent statistical analysis. Creatinine concentrations were further used to normalize the responses of features.

Before statistical analysis, the relative peak response of each metabolite at time point 1 was set to 100%. Then we compared the relative responses at time point 3 (10 h after bowel evacuation) in the bowel evacuation group (n = 6) versus the only fasting group (n = 3) using the two-tailed unpaired *t* test. *p* < 0.05 indicated that the difference was statistically significant. The heatmap of differential metabolites was obtained by Multi-Experiment viewer 4.9.0.

### 2.5. Metabolite Annotation

Metabolite annotation was firstly carried out with the OSI/SMMS software 2.4.1. In brief, the accurate MS and MS/MS spectra of ion features in analyzed samples were searched against an in-house database containing comprehensive qualitative information of more than 2000 reference chemical standards [25]. For modification-type determination of other gut microbiota-associated ion features unannotated, we used MS2Analyzer software to search for characteristic neutral losses of 40 typical metabolite modifications in human urine in the extracted MS^2^ spectra by setting parameters of the *m*/*z* window as 0.005 Da and the intensity threshold as 0.1 [26].

## 3. Results

### 3.1. Distinct Metabolites Are Decreased Subsequent to Laxative-Induced Bowel Evacuation

**Figure 1:**
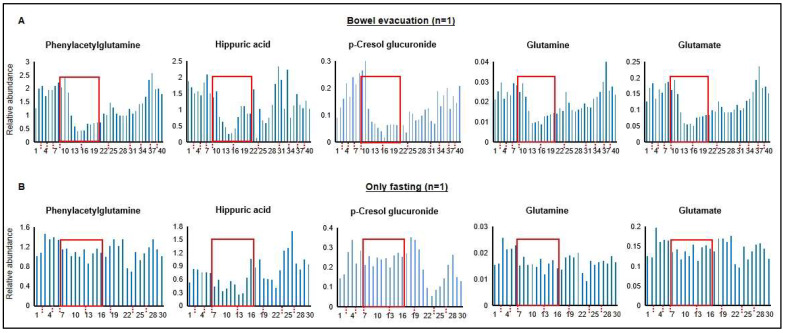
(**A**) Time courses of levels of exemplarily selected metabolites in human urine over a 10-day period before and during a laxative-induced bowel evacuation, and after starting refeeding. The red rectangles mark the 48 h period without food consumption and the dash dotted lines on the x-axes separate the different days. In total, 40 urine samples were collected (1st and 2nd morning urine, as well as spot urine) throughout the whole day (sample numbers are provided on the *x*-axes). The experiment was conducted as self-experiment from one male individual. (**B**) Control experiment, i.e., 9-day time courses of the levels of the same metabolites before and during a 48 h fasting period, and after starting refeeding. The fasting period is marked by red rectangle. In total, 30 urine samples were collected all day from the same individual. The *x*-axes show the different days and the *y*-axes the relative peak responses in arbitrary units.

Samples from the self-experiment were analyzed by non-targeted metabolomics to test our hypothesis of the detection of fecal microbiota-associated metabolites in human urine by a comparison of samples collected before and after a laxative-induced bowel evacuation. Exemplarily, Figure 1A shows at 40 different time points during a 10-day period the time courses of the levels of phenylacetylglutamine, hippuric acid, p-cresol glucuronide, glutamine, and glutamate. A persistent decrease after the bowel evacuation until the start of refeeding is clearly visible. To exclude the possibility that the detected decrease was caused by fasting, since the procedure of bowel evacuation entailed a 48 h fasting period, the same male individual performed a second self-experiment lasting 9 days, which included a “fasting-only” phase of 48 h (Figure 1B). Differences in the time courses of metabolite signal intensities between the bowel evacuation and the exclusively fasting experiment are obvious (Figure 1). Anesthesia by propofol was included in the period of the bowel evacuation, because a colonoscopy was executed in the scope of a health check. Therefore, a possible propofol effect on metabolite levels could not be excluded at this time point.

Based on this single individuum experiment, we concluded that the experimental design was suitable. In addition, possible time points for sample collection after bowel evacuation (≥10 h) could be extracted from the achieved data for subsequent studies.

### 3.2. Confirmation of the Findings of Luminal (Fecal) Microbiota-Associated Metabolites in Human Urine

**Figure 2:**
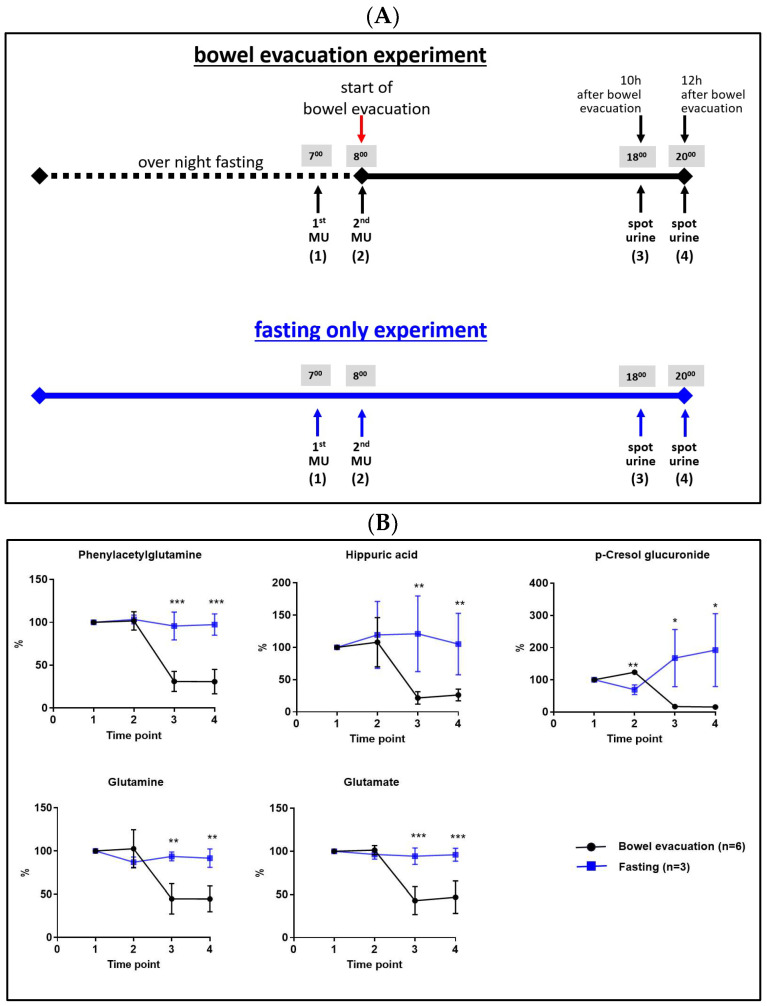
(**A**) Scheme of the experimental design and sample collection time points. (**B**) Metabolite levels in human urine collected at four time points before and after a bowel evacuation including 24 h fasting period (n = 6, black lines), and only fasting for 24 h (n = 3, blue lines). Time point 1: 1st morning urine; time point 2: 2nd morning urine, collected directly before the start of the bowel evacuation; time point 3: collected 10 h after bowel evacuation; time point 4: collected 12 h after bowel evacuation. Bars represent mean ± SD; the student’s *t*-test between groups: * *p* < 0.05, ** *p* < 0.01, *** *p* < 0.001.

Next, we aimed to (a) confirm the preceding findings, (b) exclude possible propofol effects on the findings, and (c) shorten the fasting period to adjust the sample collection to the usual procedure during a common health check colonoscopy. This laxative-induced bowel evacuation experiment was performed by six volunteers but without subsequent colonoscopy, i.e., without propofol anesthesia. The time from the start of bowel evacuation until refeeding was reduced to 12 h and the total fasting time was reduced to 24 h. Urine samples were collected at four time points. A scheme illustrating the experimental design and sample collection time points is provided in Figure 2A. Furthermore, a “fasting-only” experiment with identical sample collection time points was performed by three volunteers as a control experiment. Figure 2B shows the confirmation of all findings achieved in the n = 1 experiment (Figure 1). Based on these findings we could not only validate our preceding results, but could also exclude effects of propofol, as well as confirm the time frame of 10–12 h after bowel evacuation for sample collection as well-suited.

### 3.3. A Considerable Number of Metabolites in Human Urine Are Associated to the Luminal (Fecal) Microbiome

**Figure 3:**
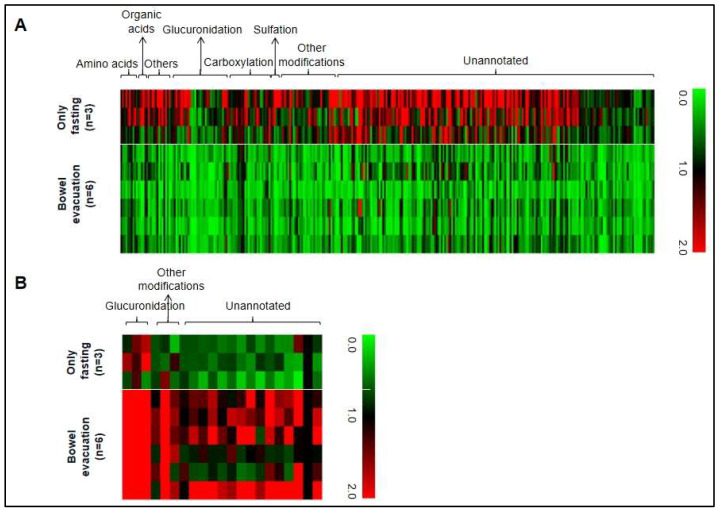
(**A**) Heat map of 310 metabolites in human urine showing significantly decreased metabolite levels after laxative-induced bowel evacuation (n = 6) in comparison to only fasting (n = 3). (**B**) Significantly increased levels of 21 metabolites after bowel evacuation (n = 6) in comparison to only fasting (n = 3). A significant difference was defined as *p* < 0.05 in a two-tailed unpaired t test comparing relative responses at time point 3 (10 h after bowel evacuation versus the only fasting group at the same time point). In the heat map, each urinary metabolite is represented by a single column. Rows represent different individuals. Black is the intensity at time point 1, green labels show decreased signal intensities, and red labels show increased signal intensities.

Next, we evaluated the data of all covered metabolite ion masses detected by non-targeted metabolomics analysis. After LC-MS data pretreatment, 7501 features remained. Around 4% (331 metabolite ion masses) were significantly altered by bowel evacuation and were therefore labeled as associated with the fecal microbiota. The majority of these metabolites were decreased after bowel evacuation, suggesting a production or transformation of these compounds by luminal gut microbes (Figure 3A). Profiling 40 different kinds of modifications led to the detection of 94 modified metabolites among these 310 fecal microbiota-associated metabolites that decreased after bowel evacuation (details in Table S2). Glucuronidation dominated these modifications (36%), followed by carboxylation (26%), sulfation (5%), adenine conjugation (4%), butyrylation (4%), malonylation (3%), acetylation (2%), and other modification types (20%). Interestingly, 21 fecal microbiome-associated metabolites, including six modified metabolite signals, were increased in comparison to the “fasting-only” control experiment (Figure 3B and Table S2), which may imply that gut microbes contribute to the suppression of their levels.

Among the 331 luminal microbiota-associated metabolite features, 32 were structurally elucidated and 6 were confirmed by standard compounds. Table 1 provides a list of annotated fecal microbiota-associated metabolites detected in human urine.

## 4. Discussion

In our study, we followed the hypothesis that gut microbiota-associated metabolites can be profiled by metabolomics investigations of urine samples. As a proof of concept, we compared the urinary metabolome before and after a bowel evacuation. We speculated that the massive reduction in the luminal (fecal) microbiota should affect the levels of associated metabolites. In Figure 1, Figure 2 and Figure 3, the association of a considerable number of metabolites with the fecal gut microbiota was demonstrated, first in a self-experiment of one male individual and then confirmed in a subsequent experiment. Furthermore, the conditions were optimized and adjusted in a way that the sample collection matched the regular procedure during a colonoscopy performed as a healthcare check, meaning two sampling time points, one just before bowel evacuation and the other 10–12 h thereafter. This could open promising perspectives for gut microbiome research studies, particularly for the generation of a fecal microbiome-associated metabolite map (M3) in urine from healthy individuals as a first step, which then could build a base for comparisons of fecal gut microbiome-associated urinary metabolome profiles in disease-related contexts in the future.

The gut microbiota consists of two fractions, namely the luminal (fecal) microbiota and mucosal microbiota. The bacterial composition, overall abundance, and diversity of luminal and mucosal microbiota vary along the longitudinal axis of the gut based on differences in environmental parameters like pH, pO2, osmolality, or mucus type. Also, the microbiota mass varies. In comparison to their sparse distribution in the small intestine, the colon is densely colonized [37] and fecal matter is the most intensely studied sample material in gut microbiome research. Recently, it was reported that fecal samples provided a good approximation of the luminal microbiome [38]. Most likely, the laxative-induced bowel evacuation applied in our study affected or reduced most of the fecal microbiome of the colon. This suggests that our findings describe foremost gut microbiome-associated metabolites in urine related to the luminal microbiota, although it was recently reported that the analysis of feces also provides a good approximation of the average gut mucosal microbiome [38].

It is well-known that gut microbiota are suitable for producing and releasing compounds which subsequently affect either positively or negatively the health state of their human host [1,2,3,4]. Furthermore, gut microbiota can introduce modifications in metabolites by numerous enzymatic reactions, thereby changing the molecular structure, chemical properties, and as a consequence, frequently also their functions. Comprehensive profiling with a relatively high certainty that the detected biomarkers originate from the gut microbiome is only suitable in feces, but not in blood or other body fluids. Consequently, until now, a considerable number of those microbiota-derived metabolites in human body fluids as well as their functions for human health or disease remain unknown. On the other hand, gut microbiota-associated metabolites may reach the liver via the splanchnic bed and the mesenteric veins and then partially filtered blood passes through the hepatic veins into the systemic circulation, including the kidneys. In the kidneys, small molecules like metabolites are filtrated into the primary urine, reach the bladder, and can finally be collected in urine samples.

Phenylacetylglutamine (PAGN), one example of the fecal gut microbiota-associated metabolites detected in urine in our study, was very recently described as a product of the interaction of intestinal microbiota and human metabolism [13]. For decades, PAGN has been recognized as a side product of phenylalanine catabolism formed in liver and renal tissues of humans and primates from phenylacetic acid [39,40]. In 2017, Dodd and colleagues showed in bacterial cultures that the PAGN precursor phenylacetic acid can also be produced by bacterial fermentation of phenylalanine [28]. The prerequisite in vivo in humans for this fermentation step is that dietary phenylalanine reaches the large intestine. Phenylalanine is then metabolized by gut microbiota to phenylpyruvic acid and subsequently to phenylacetic acid, which is taken up into the portal system [1]. Recently, PAGN has been reported to be associated with cardiovascular diseases (CVD) and incident major adverse cardiovascular events (myocardial infarction, stroke, or death) [13] as well as heart failure [41].

Hippuric acid, one of the most abundant organic acids in human urine, is derived from two different metabolic pathways; both are interplays between gut microbiota and the liver [29]. On the one hand, it can originate from phenylalanine metabolized by gut microbiota to phenylpropionic acid, which is then re-oxidized to hippuric acid involving medium-chain acyl-CoA dehydrogenase [14]. On the other hand, dietary polyphenols from fruits and vegetables like epicatechins or chlorogenic acid are metabolized by the gut microbiota to benzoic acid, which is subsequently taken up into the splanchnic bed and transported to the human liver [30]. In the liver, hippuric acid is formed by the conjugation of glycine to benzoic acid.

The production of cresol from tyrosine has been recently attributed to four intestinal strains with high cresol production activity belonging to *Coriobacteriaceae* or to *Clostridium* cluster XI or XIVa, and 55 bacterial strains were described with cresol-producing potential [31]. The cresol metabolite p-cresol glucuronide as well as the above-mentioned phenylacetylglutamine showed in plasma stronger associations with several species from the *Clostridiales* order of the corresponding gut microbiota [16].

An unexpected interesting finding of our study was the detected decrease in glutamine and glutamate after bowel evacuation, both well-known amino acids in human metabolic pathways, generated by various tissues and cells in the human body. Recently, it was demonstrated that androgen modulated circulating glutamine and that the glutamine/glutamate (Gln/Glu) ratio partially depends on the gut microbiome [3,42], and the association of metabolic disorders and diabetes with blood levels of Glu, Gln has been reported [3]. The gut microbiome can modulate brain function and behaviors through the microbiota–gut–brain axis by affecting the Glu and Gln levels, which has recently been described for example in schizophrenia [12]. In mice, probiotic treatment with *Lactobacillus rhamnosus* (JB-1) showed a significant increase in brain Glu and Gln levels [27]. Future studies to clarify the pathophysiological role of the gut microbiota-associated glutamate and glutamine metabolism for mental diseases as well as possible probiotic therapeutic options are needed.

We also detected a considerable number of modified metabolites in urine within the group of fecal gut microbiota-associated metabolites (Table S2), dominated by glucuronidations, carboxylations, and sulfations. Glucuronidation of molecules is mainly known as a detoxification reaction of endogenous and exogenous compounds in the liver; however, as shown by our data and in the literature, glucuronidation can also be gut microbiota-associated [32,43]. Recently, applying a new specific profiling strategy for carboxylations, including a derivatization step, 261 gut microbiome-associated modified metabolites were detected [44]. In various metabolite classes, carboxylated compounds have been described, like fatty acids, bile acids, *N*-acyl amino acids, benzoheterocyclic acids, or aromatic acids [44]. Sulfated metabolites are a group of modified metabolites derived from gut microbiota–human co-metabolism, which have also been reported in the context of disease development. Recently a new enzyme-assisted metabolic profiling approach reported the discovery of 206 sulfated metabolites in human feces and urine, which was three times more than the content of the commonly used Human Metabolome Database [22]. In a subsequent study, the authors showed that a polyphenol-rich diet led to an increase in the levels of 236 sulfated metabolites [45]. Interestingly, although a standardized polyphenol-rich diet was consumed, the authors observed a broad interindividual variability in the generation of these modified metabolites, which led them to speculate about high- and low-sulfate metabolizers [45]. Metabolites modified by gut microbiota could be an interesting additional class of metabolites in biomedical research, but they often remain until now unknown in metabolomics datasets, since they are still underrepresented in all common big databases.

A potential weakness with respect to the data presented here is that in our approach with laxative use, only the luminal (fecal) microbiome in the gut is massively reduced, but no total eradication of the gut microbiome (luminal and mucosal) was achieved, unless antibiotics were used. Consequently, gut microbiota-associated metabolites of distinct species or maybe also phyla may not be detectable with our strategy. However, it was recently reported that fecal samples provide a good approximation of the luminal as well as of the average gut mucosal microbiome [38]. Furthermore, since urine was investigated as a non-invasively collected sample material, the covered fecal microbiota-associated metabolites were limited to water-soluble compounds, which were either per se more or less polar or which were modified before urinary excretion by the liver, e.g., by conjugation of glucuronides. Hence, water-insoluble, apolar gut microbiome-associated metabolites, which can only be detected by the investigation of feces, or in body fluids by invasive sampling (e.g., in blood samples), were not covered. Concerning sex differences in the gut microbiome, which are under intense discussion and investigation [46], we cannot draw any conclusions on the metabolite level based on our data.

## 5. Conclusions

Beginning with a single-individual self-experiment for hypothesis testing, which covered 40 sample collection time points spreading over 10 days, we revealed gut microbiota association of metabolites in urine after the depletion of the fecal microbiome by a laxative-induced bowel evacuation. These findings were confirmed in a subsequent study performed in six individuals with four sample collection time points by the detection of numerous fecal microbiota-associated metabolites, including modified metabolites. We conclude that our strategy is suitable to profile luminal or fecal microbiome-associated metabolites in urine and consequently detect, e.g., disease-related differences in human gut microbiomes, as well as therapy-dependent changes. Overall, our strategy opens new perspectives for comprehensive human microbiome studies in biomedical research and beyond.

## Figures and Tables

**Table 1:** Annotated fecal microbiota-associated metabolites. Identity is either based on confirmation with a standard compound or putative annotation based on exact mass (MS^1^) and fragmentation patterns (MS^2^).

No.	Metabolites	Annotation Base	Category	Selected Microbiota-Related References
1	Phenylalanine	a, b, c	Amino acid	[8]
2	Glutamine	a, b	Amino acid	[12,27]
3	Glutamate	a, b	Amino acid	[12,27]
4	Methionine	a, b	Amino acid	[8]
5	Tryptophan	a, b	Amino acid	[8]
6	*N*-Acetyltryptophan	a, b	Amino acid	[8]
7	5-Hydroxytryptophan	a, b	Amino acid	[8]
8	*N*-Acetyltyrosine	a, b	Amino acid	[8]
9	*N*-(3-Indolylacetyl)-l-alanine	a, b	Amino acid	[8]
10	*N*-cyclohexyltaurine	a, b	Amino acid	
11	Phenylacetylglutamine	a, b, c	Amino acid	[13,28]
12	Hippuric acid	a, b, c	Organic acid	[29,30]
13	Hydroxyhippuric acid	a, b	Organic acid	[8]
14	Hydroxyphenyl lactic acid	a, b	Organic acid	[8]
15	5-Hydroxyindole-3-acetic acid	a, b	Organic acid	[8]
16	Aminobutyric acid	a, b, c	Organic acid	[8]
17	Dimethyluric acid	a, b, c	Organic acid	[8]
18	Aminooctanoic acid	a, b	Organic acid	[8]
19	p-Cresol glucuronide	a, b	Organic acid	[16,31,32]
20	Dimethylxanthine	a, b	Nucleoside	[33]
21	Orotidine	a, b	Nucleoside	[8]
22	8-Hydroxy-2-deoxyguanosine	a, b	Nucleoside	
23	Decanoylcarnitine	a, b, c	Others	[8]
24	Tyrosol	a, b	Others	[34]
25	Hydroxybenzyl alcohol	a, b	Others	
26	2-Methyl-1,2,3,4-tetrahydro-6,7-isoquinolinediol	a, b	Others	
27	4-Hydroxyquinoline	a, b	Others	[8]
28	Hydroxybenzaldehyde	a, b	Others	[35]
29	Dihydroxyacetone	a, b	Others	[36]
30	Acetamidophenyl glucuronide	a, b	Others	[32]
31	3-Methyloxindole	a, b	Others	[8]
32	Phenylacetamide	a, b	Others	[8]

a: exact mass, b: MS/MS spectra, c: confirmed by a standard.

## Data Availability

The data presented in this study are available on request from the corresponding author. The data are not publicly available due to privacy restrictions.

## Supplementary Materials

The following supporting information can be downloaded at: https://www.mdpi.com/article/10.3390/metabo13101061/s1, Table S1: Detailed information of internal standards; Table S2: Detailed information about specific modifying groups of gut microbiota-associated metabolite features.

## References

[1] Gentile C.L., Weir T.L. (2018). The gut microbiota at the intersection of diet and human health. Science.

[2] Selber-Hnatiw S., Sultana T., Tse W., Abdollahi N., Abdullah S., Al Rahbani J., Alazar D., Alrumhein N.J., Aprikian S., Arshad R. (2020). Metabolic networks of the human gut microbiota. Microbiology.

[3] Liu R., Hong J., Xu X., Feng Q., Zhang D., Gu Y., Shi J., Zhao S., Liu W., Wang X. (2017). Gut microbiome and serum metabolome alterations in obesity and after weight-loss intervention. Nat. Med..

[4] Fan Y., Pedersen O. (2021). Gut microbiota in human metabolic health and disease. Nat. Rev. Microbiol..

[5] Hills R.D., Pontefract B.A., Mishcon H.R., Black C.A., Sutton S.C., Theberge C.R. (2019). Gut Microbiome: Profound Implications for Diet and Disease. Nutrients.

[6] Whon T.W., Shin N.R., Kim J.Y., Roh S.W. (2021). Omics in gut microbiome analysis. J. Microbiol..

[7] Manor O., Dai C.L., Kornilov S.A., Smith B., Price N.D., Lovejoy J.C., Gibbons S.M., Magis A.T. (2020). Health and disease markers correlate with gut microbiome composition across thousands of people. Nat. Commun..

[8] Han S., Van Treuren W., Fischer C.R., Merrill B.D., DeFelice B.C., Sanchez J.M., Higginbottom S.K., Guthrie L., Fall L.A., Dodd D. (2021). A metabolomics pipeline for the mechanistic interrogation of the gut microbiome. Nature.

[9] Wu H., Tremaroli V., Schmidt C., Lundqvist A., Olsson L.M., Kramer M., Gummesson A., Perkins R., Bergstrom G., Backhed F. (2020). The Gut Microbiota in Prediabetes and Diabetes: A Population-Based Cross-Sectional Study. Cell Metab..

[10] David L.A., Maurice C.F., Carmody R.N., Gootenberg D.B., Button J.E., Wolfe B.E., Ling A.V., Devlin A.S., Varma Y., Fischbach M.A. (2014). Diet rapidly and reproducibly alters the human gut microbiome. Nature.

[11] Li M., Wang B., Zhang M., Rantalainen M., Wang S., Zhou H., Zhang Y., Shen J., Pang X., Zhang M. (2008). Symbiotic gut microbes modulate human metabolic phenotypes. Proc. Natl. Acad. Sci. USA.

[12] Zheng P., Zeng B., Liu M., Chen J., Pan J., Han Y., Liu Y., Cheng K., Zhou C., Wang H. (2019). The gut microbiome from patients with schizophrenia modulates the glutamate-glutamine-GABA cycle and schizophrenia-relevant behaviors in mice. Sci. Adv..

[13] Nemet I., Saha P.P., Gupta N., Zhu W., Romano K.A., Skye S.M., Cajka T., Mohan M.L., Li L., Wu Y. (2020). A Cardiovascular Disease-Linked Gut Microbial Metabolite Acts via Adrenergic Receptors. Cell.

[14] Pruss K.M., Chen H., Liu Y., Van Treuren W., Higginbottom S.K., Jarman J.B., Fischer C.R., Mak J., Wong B., Cowan T.M. (2023). Host-microbe co-metabolism via MCAD generates circulating metabolites including hippuric acid. Nat. Commun..

[15] Kikuchi K., Saigusa D., Kanemitsu Y., Matsumoto Y., Thanai P., Suzuki N., Mise K., Yamaguchi H., Nakamura T., Asaji K. (2019). Gut microbiome-derived phenyl sulfate contributes to albuminuria in diabetic kidney disease. Nat. Commun..

[16] Dekkers K.F., Sayols-Baixeras S., Baldanzi G., Nowak C., Hammar U., Nguyen D., Varotsis G., Brunkwall L., Nielsen N., Eklund A.C. (2022). An online atlas of human plasma metabolite signatures of gut microbiome composition. Nat. Commun..

[17] Zierer J., Jackson M.A., Kastenmuller G., Mangino M., Long T., Telenti A., Mohney R.P., Small K.S., Bell J.T., Steves C.J. (2018). The fecal metabolome as a functional readout of the gut microbiome. Nat. Genet..

[18] Hu J., Ding J., Li X., Li J., Zheng T., Xie L., Li C., Tang Y., Guo K., Huang J. (2023). Distinct signatures of gut microbiota and metabolites in different types of diabetes: A population-based cross-sectional study. EClinicalMedicine.

[19] Hryhorczuk L.M., Novak E.A., Gershon S. (1984). Gut flora and urinary phenylacetic acid. Science.

[20] Goodwin B.L., Ruthven C.R., Sandler M. (1994). Gut flora and the origin of some urinary aromatic phenolic compounds. Biochem. Pharmacol..

[21] Li R.J., Jie Z.Y., Feng Q., Fang R.L., Li F., Gao Y., Xia H.H., Zhong H.Z., Tong B., Madsen L. (2021). Network of Interactions between Gut Microbiome, Host Biomarkers, and Urine Metabolome in Carotid Atherosclerosis. Front. Cell Infect. Microbiol..

[22] Ballet C., Correia M.S.P., Conway L.P., Locher T.L., Lehmann L.C., Garg N., Vujasinovic M., Deindl S., Lohr J.M., Globisch D. (2018). New enzymatic and mass spectrometric methodology for the selective investigation of gut microbiota-derived metabolites. Chem. Sci..

[23] Jain A., Li X.H., Chen W.N. (2019). An untargeted fecal and urine metabolomics analysis of the interplay between the gut microbiome, diet and human metabolism in Indian and Chinese adults. Sci. Rep..

[24] Yang J., Zhao X., Lu X., Lin X., Xu G. (2015). A data preprocessing strategy for metabolomics to reduce the mask effect in data analysis. Front. Mol. Biosci..

[25] Zhao X., Zeng Z., Chen A., Lu X., Zhao C., Hu C., Zhou L., Liu X., Wang X., Hou X. (2018). Comprehensive Strategy to Construct In-House Database for Accurate and Batch Identification of Small Molecular Metabolites. Anal. Chem..

[26] Zheng S., Zhang X., Li Z., Hoene M., Fritsche L., Zheng F., Li Q., Fritsche A., Peter A., Lehmann R. (2021). Systematic, Modifying Group-Assisted Strategy Expanding Coverage of Metabolite Annotation in Liquid Chromatography-Mass Spectrometry-Based Nontargeted Metabolomics Studies. Anal. Chem..

[27] Janik R., Thomason L.A.M., Stanisz A.M., Forsythe P., Bienenstock J., Stanisz G.J. (2016). Magnetic resonance spectroscopy reveals oral Lactobacillus promotion of increases in brain GABA, N-acetyl aspartate and glutamate. Neuroimage.

[28] Dodd D., Spitzer M.H., Van Treuren W., Merrill B.D., Hryckowian A.J., Higginbottom S.K., Le A., Cowan T.M., Nolan G.P., Fischbach M.A. (2017). A gut bacterial pathway metabolizes aromatic amino acids into nine circulating metabolites. Nature.

[29] Ticinesi A., Guerra A., Nouvenne A., Meschi T., Maggi S. (2023). Disentangling the Complexity of Nutrition, Frailty and Gut Microbial Pathways during Aging: A Focus on Hippuric Acid. Nutrients.

[30] Penczynski K.J., Krupp D., Bring A., Bolzenius K., Remer T., Buyken A.E. (2017). Relative validation of 24-h urinary hippuric acid excretion as a biomarker for dietary flavonoid intake from fruit and vegetables in healthy adolescents. Eur. J. Nutr..

[31] Saito Y., Sato T., Nomoto K., Tsuji H. (2018). Identification of phenol- and p-cresol-producing intestinal bacteria by using media supplemented with tyrosine and its metabolites. FEMS Microbiol. Ecol..

[32] Pellock S.J., Redinbo M.R. (2017). Glucuronides in the gut: Sugar-driven symbioses between microbe and host. J. Biol. Chem..

[33] Yue S., Zhao D., Peng C., Tan C., Wang Q., Gong J. (2019). Effects of theabrownin on serum metabolites and gut microbiome in rats with a high-sugar diet. Food Funct..

[34] Mosele J.I., Martin-Pelaez S., Macia A., Farras M., Valls R.M., Catalan U., Motilva M.J. (2014). Faecal microbial metabolism of olive oil phenolic compounds: In vitro and in vivo approaches. Mol. Nutr. Food Res..

[35] Li Y., Sui L., Zhao H., Zhang W., Gao L., Hu W., Song M., Liu X., Kong F., Gong Y. (2022). Differences in the Establishment of Gut Microbiota and Metabolome Characteristics between Balb/c and C57BL/6J Mice after Proton Irradiation. Front. Microbiol..

[36] Jamshidi N., Nigam S.K. (2022). Drug transporters OAT1 and OAT3 have specific effects on multiple organs and gut microbiome as revealed by contextualized metabolic network reconstructions. Sci. Rep..

[37] McCallum G., Tropini C. (2023). The gut microbiota and its biogeography. Nat. Rev. Microbiol..

[38] Vaga S., Lee S., Ji B., Andreasson A., Talley N.J., Agreus L., Bidkhori G., Kovatcheva-Datchary P., Park J., Lee D. (2020). Compositional and functional differences of the mucosal microbiota along the intestine of healthy individuals. Sci. Rep..

[39] Moldave K., Meister A. (1957). Synthesis of phenylacetylglutamine by human tissue. J. Biol. Chem..

[40] Yang D., Brunengraber H. (2000). Glutamate, a window on liver intermediary metabolism. J. Nutr..

[41] Romano K.A., Nemet I., Prasad Saha P., Haghikia A., Li X.S., Mohan M.L., Lovano B., Castel L., Witkowski M., Buffa J.A. (2023). Gut Microbiota-Generated Phenylacetylglutamine and Heart Failure. Circ. Heart Fail..

[42] Gao A., Su J., Liu R., Zhao S., Li W., Xu X., Li D., Shi J., Gu B., Zhang J. (2021). Sexual dimorphism in glucose metabolism is shaped by androgen-driven gut microbiome. Nat. Commun..

[43] Wikoff W.R., Anfora A.T., Liu J., Schultz P.G., Lesley S.A., Peters E.C., Siuzdak G. (2009). Metabolomics analysis reveals large effects of gut microflora on mammalian blood metabolites. Proc. Natl. Acad. Sci. USA.

[44] Wang Y.Z., Chen Y.Y., Wu X.Z., Bai P.R., An N., Liu X.L., Zhu Q.F., Feng Y.Q. (2023). Uncovering the Carboxylated Metabolome in Gut Microbiota-Host Co-metabolism: A Chemical Derivatization-Molecular Networking Approach. Anal. Chem..

[45] Correia M.S.P., Jain A., Alotaibi W., Young Tie Yang P., Rodriguez-Mateos A., Globisch D. (2020). Comparative dietary sulfated metabolome analysis reveals unknown metabolic interactions of the gut microbiome and the human host. Free Radic. Biol. Med..

[46] Valeri F., Endres K. (2021). How biological sex of the host shapes its gut microbiota. Front. Neuroendocrinol..

