# Supplementary material for: A New Biomarker Profiling Strategy for Gut Microbiome Research: Valid Association of Metabolites to Metabolism of Microbiota Detected by Non-Targeted Metabolomics in Human Urine
Source: Metabolites. 2023 Oct 9;13(10):1061. doi: 10.3390/metabo13101061 (PMC10608496; doi:10.3390/metabo13101061)
Supplement: Supplementary file 1 [file metabolites-13-01061-s001.zip › metabolites-2629586-supplementary.pdf]

Supporting Information

Table S1. Detailed information of internal standards (ISs).

IS	Solvent	Concentration in IS-mix (µg/ml)
Carnitine C2:0-d3	100% Methanol	41.01
Carnitine C6:0-d3	100% Methanol	3.48
Carnitine C10:0-d3	100% Methanol	3.75
Leucine-d3	HPLC-water	44.4
Phenylalanine-d5	HPLC-water	17.25
Tryptophan-d5	HPLC-water	34.5
Cholic acid-2,2,4,4-d4	100% Methanol	7.5
Chenodeoxycholic acid-2,2,4,4-d4	100% Methanol	8.1
Leucine Enkephalin	HPLC-water	18
Indoxyl sulfate-[$^{13}C_6$]	HPLC-water	60
L-Valine-d8, 98%	HPLC-water	60
Sodium-2-Hydroxybutyrate-2,3,3-d3	100% Methanol	60
L-4-Hydroxyphenyl-d4-alanine	HPLC-water	60

Table S2. Detailed information about specific modifying groups of the detected gut microbiota-associated modified metabolite features.

No.	Modification	Detection mode	m/z	t_R/min	Fold change (bowel evacuation/fasting only)
1	Glucuronidation	Neg	509.2377	15.22	0.51
2	Glucuronidation	Neg	497.2388	7.93	0.05
3	Glucuronidation	Neg	476.1934	12.70	0.32
4	Glucuronidation	Pos	470.1983	6.97	0.34
5	Glucuronidation	Neg	338.0888	2.26	0.11
6	Glucuronidation	Neg	326.0531	1.60	0.25
7	Glucuronidation	Neg	324.0731	1.35	0.23
8	Glucuronidation	Neg	303.1091	7.65	0.30
9	Glucuronidation	Neg	285.0608	2.13	0.20
10	Glucuronidation	Neg	283.0821	6.48	0.10
11	Glucuronidation	Pos	595.2520	9.93	0.19
12	Glucuronidation	Pos	595.2483	10.33	0.21
13	Glucuronidation	Neg	593.2340	9.94	0.18
14	Glucuronidation	Neg	537.2328	12.60	0.41
15	Glucuronidation	Neg	522.2356	11.49	0.50
16	Glucuronidation	Pos	480.2199	12.74	0.44
17	Glucuronidation	Pos	478.2066	12.16	0.30
18	Glucuronidation	Pos	478.2061	12.69	0.37
19	Glucuronidation	Neg	478.2061	12.76	0.24
20	Glucuronidation	Neg	476.1941	12.15	0.23
21	Glucuronidation	Pos	454.2060	10.56	0.25
22	Glucuronidation	Neg	452.1919	10.57	0.33
23	Glucuronidation	Neg	452.1918	10.22	0.39
24	Glucuronidation	Neg	437.1805	11.47	0.10
25	Glucuronidation	Neg	437.1084	9.62	0.28
26	Glucuronidation	Pos	328.1009	4.19	0.35
27	Glucuronidation	Pos	326.0876	1.34	0.40
28	Glucuronidation	Pos	326.0853	4.19	0.37
29	Glucuronidation	Neg	383.0055	6.48	0.14
30	Glucuronidation	Neg	357.0826	2.80	0.59
31	Glucuronidation	Neg	349.1507	6.90	0.50
32	Glucuronidation	Neg	427.1583	10.28	0.36
33	Glucuronidation	Pos	531.2788	11.44	0.29
34	Glucuronidation	Pos	455.0483	1.12	0.64
35	Carboxylation	Pos	567.1758	5.03	0.27
36	Carboxylation	Pos	553.1881	5.00	0.29
37	Carboxylation	Pos	383.0801	4.81	0.29

No.	Modification	Detection mode	m/z	t_R/min	Fold change (bowel evacuation/fasting only)
38	Carboxylation	Pos	335.0833	5.06	0.65
39	Carboxylation	Pos	335.0791	4.98	0.60
40	Carboxylation	Pos	307.0884	5.02	0.66
41	Carboxylation	Neg	242.1754	13.35	0.89
42	Carboxylation	Neg	180.0547	4.84	0.24
43	Carboxylation	Neg	142.0679	8.18	0.17
44	Carboxylation	Neg	190.0509	5.89	0.12
45	Carboxylation	Pos	180.0098	1.81	0.34
46	Carboxylation	Pos	147.0436	6.31	0.28
47	Carboxylation	Pos	274.1196	3.02	0.25
48	Carboxylation	Pos	206.0816	7.36	0.40
49	Carboxylation	Pos	165.0531	3.63	0.23
50	Carboxylation	Pos	152.0712	4.85	0.24
51	Carboxylation	Pos	174.0536	5.87	0.20
52	Carboxylation	Pos	130.0475	3.19	0.63
53	Carboxylation	Neg	626.1325	4.99	0.39
54	Carboxylation	Neg	363.0280	5.01	0.43
55	Carboxylation	Neg	277.9777	4.80	0.54
56	Carboxylation	Neg	253.1095	12.24	0.28
57	Carboxylation	Pos	412.0340	4.79	0.46
58	Carboxylation	Pos	303.0724	4.98	0.35
59	Sulfation	Neg	343.9294	3.63	0.04
60	Sulfation	Neg	230.0132	5.06	0.05
61	Sulfation	Neg	229.9774	3.62	0.18
62	Sulfation	Neg	227.9977	3.86	0.13
63	Sulfation	Neg	188.0023	1.88	0.20
64	Adenine conjugation	Neg	264.0894	6.29	0.30
65	Adenine conjugation	Neg	245.0936	5.03	0.27
66	Adenine conjugation	Pos	290.1049	11.61	0.46
67	Adenine conjugation	Pos	269.0592	1.81	0.42
68	Butyrylation	Pos	156.1377	6.57	0.36
69	Butyrylation	Neg	127.0531	5.00	0.30
70	Butyrylation	Pos	215.1753	5.58	0.23
71	Butyrylation	Pos	188.1262	5.09	0.42
72	Malonylation	Neg	215.1045	1.40	0.51
73	Malonylation	Neg	164.9572	0.70	0.41
74	Malonylation	Pos	201.0541	11.60	0.45
75	Acetylation	Neg	200.1293	11.39	0.45
76	Acetylation	Pos	338.1968	7.63	0.19
77	Glutathione conjugation	Pos	846.2472	5.01	0.15

No.	Modification	Detection mode	m/z	t_R/min	Fold change (bowel evacuation/fasting only)
78	Glutathione conjugation	Pos	281.1130	3.20	0.39
79	Glycine conjugation	Pos	228.0627	8.22	0.12
80	Glycine conjugation	Pos	162.0540	4.81	0.22
81	Indoleacetyl conjugation	Pos	416.1503	4.99	0.21
82	Indoleacetyl conjugation	Pos	287.0981	4.97	0.39
83	N-acetylcysteine conjugation	Pos	468.1380	4.91	0.20
84	N-acetylcysteine conjugation	Pos	311.0690	4.33	0.32
85	Phosphorylation	Neg	840.2451	8.52	0.38
86	Phosphorylation	Neg	424.0556	4.20	0.10
87	Propionylation	Pos	147.0765	4.97	0.50
88	Propionylation	Pos	107.0494	4.56	0.54
89	Anhydroaminodeoxyhexose conjugation	Pos	304.1161	12.21	0.33
90	Anhydrohexose conjugation	Neg	350.0551	3.29	0.36
91	Anhydropentose conjugation	Neg	490.8352	0.68	0.47
92	Cysteine conjugation	Pos	426.2018	9.21	0.09
93	Succinylation	Neg	243.0787	8.19	0.14
94	γ-GluCys conjugation	Neg	428.0040	6.48	0.11
95	Glucuronidation	Pos	454.1484	3.90	179.32
96	Glucuronidation	Pos	484.1585	4.38	70.31
97	Glucuronidation	Pos	514.1702	5.29	21.21
98	Acetylation	Pos	232.1291	1.43	1.61
99	Adenine conjugation	Pos	392.1536	7.47	3.16
100	Sulfation	Neg	337.9407	3.72	2.03
